# 5-Bromo-1-(prop-2-en-1-yl)-2,3-dihydro-1*H*-indole-2,3-dione

**DOI:** 10.1107/S160053681105447X

**Published:** 2011-12-23

**Authors:** Khalil Maamri, Hafid Zouihri, El Mokhtar Essassi, Seik Weng Ng

**Affiliations:** a Laboratoire de Chimie Organique Hétérocyclique, Pôle de Compétences Pharmacochimie, Université Mohammed V-Agdal, BP 1014 Avenue Ibn Batout, Rabat, Morocco; b CNRST Division UATRS, Angle Allal Fassi/FAR, BP 8027 Hay Riad, Rabat, Morocco; c Department of Chemistry, University of Malaya, 50603 Kuala Lumpur, Malaysia; d Chemistry Department, King Abdulaziz University, PO Box 80203 Jeddah, Saudi Arabia

## Abstract

In the title compound, C_11_H_8_BrNO_2_, the nine-membered fused-ring is nearly planar [maximum deviation = 0.022 (2) Å] and the allyl group is arched over the nine-membered fused-ring at a dihedral angle of 89.2 (1)°. Weak inter­molecular C—H⋯O hydrogen bonding is present in the crystal structure.

## Related literature

For a related mol­ecule, see: Abdel-Hamid *et al.* (2009 ▶).
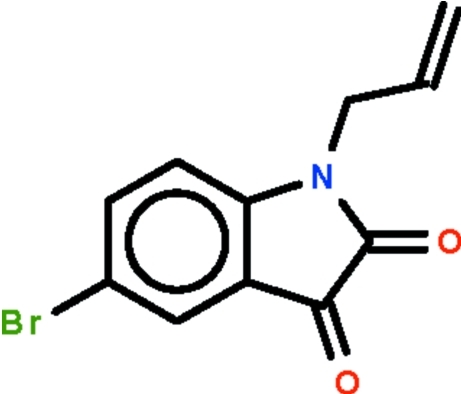


## Experimental

### 

#### Crystal data

C_11_H_8_BrNO_2_
*M* *_r_* = 266.09
Orthorhombic, 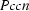

*a* = 31.3411 (5) Å
*b* = 7.8995 (1) Å
*c* = 8.2716 (1) Å
*V* = 2047.87 (5) Å^3^
*Z* = 8
Mo *K*α radiation
μ = 3.99 mm^−1^
*T* = 293 K
0.17 × 0.14 × 0.13 mm

#### Data collection

Bruker APEX DUO diffractometer
Absorption correction: multi-scan (*SADABS*; Sheldrick, 1996 ▶) *T*_min_ = 0.550, *T*_max_ = 0.625
50850 measured reflections
2983 independent reflections
2345 reflections with *I* > 2σ(*I*)
*R*_int_ = 0.035

#### Refinement

*R*[*F* ^2^ > 2σ(*F* ^2^)] = 0.029
*wR*(*F* ^2^) = 0.091
*S* = 1.06
2983 reflections
136 parameters
H-atom parameters constrained
Δρ_max_ = 0.43 e Å^−3^
Δρ_min_ = −0.63 e Å^−3^

### 

Data collection: *APEX2* (Bruker, 2010 ▶); cell refinement: *SAINT* (Bruker, 2010 ▶); data reduction: *SAINT*; program(s) used to solve structure: *SHELXS97* (Sheldrick, 2008 ▶); program(s) used to refine structure: *SHELXL97* (Sheldrick, 2008 ▶); molecular graphics: *X-SEED* (Barbour, 2001 ▶); software used to prepare material for publication: *publCIF* (Westrip, 2010 ▶).

## Supplementary Material

Crystal structure: contains datablock(s) global, I. DOI: 10.1107/S160053681105447X/xu5413sup1.cif

Structure factors: contains datablock(s) I. DOI: 10.1107/S160053681105447X/xu5413Isup2.hkl

Supplementary material file. DOI: 10.1107/S160053681105447X/xu5413Isup3.cml

Additional supplementary materials:  crystallographic information; 3D view; checkCIF report

## Figures and Tables

**Table 1:** Hydrogen-bond geometry (Å, °)

*D*—H⋯*A*	*D*—H	H⋯*A*	*D*⋯*A*	*D*—H⋯*A*
C2—H2⋯O1^i^	0.93	2.41	3.273 (2)	154
C11—H11*A*⋯O2^ii^	0.93	2.46	3.358 (3)	163

Symmetry codes: (i) 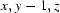; (ii) 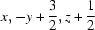.

**Fig. 1.**
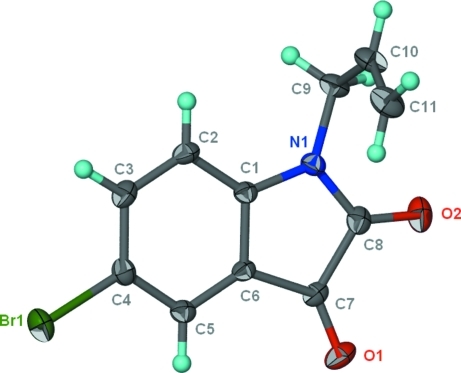
Thermal ellipsoid plot (Barbour, 2001) of C11H8BrNO2 at the 50% probability level; hydrogen atoms are drawn as spheres of arbitrary radius.

## supplementary crystallographic information

### Comment

We are interested in the pharmaceutical properites of isatin derivatives; the allyl group 1-(prop-2-en-1-yl)-2,3-dihydro-1*H*-indole-2,3-dione, whose crystal structure was recently reported (Abdel-Hamid *et al.*, 2009), is a substituent that can undergo a variety of chemical transformation. The bromo-substituted title compound (Scheme I) features a planar fused-ring; the allyl group is arched over the five-membered ring (dihedral angle between allyl plane and nine-membered fused-ring 89.2 (1)°) (Fig. 1).

### Experimental

To a solution of 5-bromo-isatin (1g, 4.4 mmole) in *N*,*N*-dimethylformamide (50 ml) was added allyl bromide (1.50 g, 12.5 mmol) potassium carbonate (1 g, 7.4 mmol) along with a catalytic quantity of tetra-*n*-butylammonium bromide. The mixture was stirred for 48 h. The reaction was monitored by thin layer chromatography. The mixture was filtered and the solution evaporated under vacuum. The solid residue was recrystallized from ethanol to afford the title compound as red crystals.

### Refinement

Carbon-bound H-atoms were placed in calculated positions (C–H 0.93–0.97 Å) and were included in the refinement in the riding model approximation, with *U*(H) set to 1.2*U*(C).

Omitted was the 2 0 0 reflection.

### Crystal data

**Table:**

C_11_H_8_BrNO_2_	*F*(000) = 1056
*M**_r_* = 266.09	*D*_x_ = 1.726 Mg m^−^^3^
Orthorhombic, *P**c**c**n*	Mo *K*α radiation, λ = 0.71073 Å
Hall symbol: -P 2ab 2ac	Cell parameters from 9894 reflections
*a* = 31.3411 (5) Å	θ = 2.6–31.7°
*b* = 7.8995 (1) Å	µ = 3.99 mm^−^^1^
*c* = 8.2716 (1) Å	*T* = 293 K
*V* = 2047.87 (5) Å^3^	Prism, red
*Z* = 8	0.17 × 0.14 × 0.13 mm

### Data collection

**Table:**

Bruker APEX DUO diffractometer	2983 independent reflections
Radiation source: fine-focus sealed tube	2345 reflections with *I* > 2σ(*I*)
graphite	*R*_int_ = 0.035
ω scans	θ_max_ = 30.0°, θ_min_ = 2.6°
Absorption correction: multi-scan (*SADABS*; Sheldrick, 1996)	*h* = −44→44
*T*_min_ = 0.550, *T*_max_ = 0.625	*k* = −6→11
50850 measured reflections	*l* = −11→11

### Refinement

**Table:**

Refinement on *F*^2^	Primary atom site location: structure-invariant direct methods
Least-squares matrix: full	Secondary atom site location: difference Fourier map
*R*[*F*^2^ > 2σ(*F*^2^)] = 0.029	Hydrogen site location: inferred from neighbouring sites
*wR*(*F*^2^) = 0.091	H-atom parameters constrained
*S* = 1.06	*w* = 1/[σ^2^(*F*_o_^2^) + (0.0456*P*)^2^ + 1.4798*P*] where *P* = (*F*_o_^2^ + 2*F*_c_^2^)/3
2983 reflections	(Δ/σ)_max_ = 0.001
136 parameters	Δρ_max_ = 0.43 e Å^−^^3^
0 restraints	Δρ_min_ = −0.63 e Å^−^^3^

### Fractional atomic coordinates and isotropic or equivalent isotropic displacement parameters (Å^2^)

**Table:**

	*x*	*y*	*z*	*U*_iso_*/*U*_eq_	
Br1	0.241206 (6)	0.49599 (3)	0.59089 (3)	0.03615 (9)	
O1	0.12709 (5)	0.94850 (17)	0.22127 (18)	0.0334 (3)	
O2	0.06568 (4)	0.77943 (19)	0.00288 (18)	0.0360 (3)	
N1	0.09810 (5)	0.5408 (2)	0.10576 (18)	0.0237 (3)	
C1	0.13156 (5)	0.5079 (2)	0.2140 (2)	0.0204 (3)	
C2	0.14721 (6)	0.3526 (2)	0.2635 (2)	0.0253 (3)	
H2	0.1358	0.2520	0.2242	0.030*	
C3	0.18068 (6)	0.3522 (2)	0.3744 (2)	0.0270 (4)	
H3	0.1919	0.2497	0.4098	0.032*	
C4	0.19752 (6)	0.5033 (2)	0.4327 (2)	0.0250 (3)	
C5	0.18246 (5)	0.6597 (2)	0.3816 (2)	0.0229 (3)	
H5	0.1943	0.7602	0.4192	0.027*	
C6	0.14914 (5)	0.6589 (2)	0.2724 (2)	0.0200 (3)	
C7	0.12453 (5)	0.7978 (2)	0.2011 (2)	0.0229 (3)	
C8	0.09146 (5)	0.7101 (2)	0.0887 (2)	0.0256 (4)	
C9	0.07183 (6)	0.4118 (2)	0.0275 (2)	0.0295 (4)	
H9A	0.0894	0.3138	0.0044	0.035*	
H9B	0.0615	0.4560	−0.0748	0.035*	
C10	0.03449 (6)	0.3572 (3)	0.1269 (3)	0.0342 (4)	
H10	0.0177	0.2706	0.0850	0.041*	
C11	0.02314 (7)	0.4189 (3)	0.2666 (3)	0.0379 (5)	
H11A	0.0389	0.5057	0.3135	0.046*	
H11B	−0.0008	0.3762	0.3193	0.046*	

### Atomic displacement parameters (Å^2^)

**Table:**

	*U*^11^	*U*^22^	*U*^33^	*U*^12^	*U*^13^	*U*^23^
Br1	0.02822 (13)	0.04590 (15)	0.03435 (13)	0.00097 (8)	−0.00721 (7)	0.00370 (8)
O1	0.0391 (8)	0.0187 (6)	0.0424 (8)	0.0006 (6)	0.0109 (6)	0.0018 (6)
O2	0.0273 (6)	0.0407 (8)	0.0400 (8)	0.0049 (6)	−0.0013 (6)	0.0119 (6)
N1	0.0215 (7)	0.0233 (7)	0.0264 (7)	−0.0038 (6)	−0.0012 (6)	−0.0004 (6)
C1	0.0190 (7)	0.0200 (7)	0.0223 (7)	−0.0026 (6)	0.0031 (6)	−0.0009 (6)
C2	0.0287 (9)	0.0173 (7)	0.0300 (9)	−0.0020 (6)	0.0035 (7)	−0.0015 (6)
C3	0.0280 (8)	0.0237 (8)	0.0292 (8)	0.0033 (7)	0.0042 (7)	0.0033 (7)
C4	0.0205 (7)	0.0303 (9)	0.0240 (8)	−0.0001 (6)	0.0014 (6)	0.0017 (7)
C5	0.0215 (7)	0.0233 (8)	0.0238 (8)	−0.0034 (6)	0.0037 (6)	−0.0021 (6)
C6	0.0191 (7)	0.0167 (7)	0.0241 (8)	−0.0021 (6)	0.0054 (6)	−0.0009 (6)
C7	0.0233 (8)	0.0193 (7)	0.0261 (8)	−0.0006 (6)	0.0087 (6)	0.0013 (6)
C8	0.0203 (7)	0.0282 (8)	0.0283 (9)	−0.0002 (6)	0.0059 (6)	0.0041 (7)
C9	0.0274 (9)	0.0330 (9)	0.0280 (9)	−0.0077 (7)	−0.0016 (7)	−0.0066 (7)
C10	0.0303 (9)	0.0370 (10)	0.0353 (10)	−0.0142 (8)	−0.0030 (8)	−0.0007 (8)
C11	0.0307 (10)	0.0483 (13)	0.0348 (10)	−0.0133 (9)	0.0031 (8)	0.0024 (9)

### Geometric parameters (Å, °)

**Table:**

Br1—C4	1.8950 (19)	C4—C5	1.388 (2)
O1—C7	1.205 (2)	C5—C6	1.381 (2)
O2—C8	1.207 (2)	C5—H5	0.9300
N1—C8	1.361 (2)	C6—C7	1.465 (2)
N1—C1	1.403 (2)	C7—C8	1.555 (3)
N1—C9	1.461 (2)	C9—C10	1.494 (3)
C1—C2	1.383 (2)	C9—H9A	0.9700
C1—C6	1.400 (2)	C9—H9B	0.9700
C2—C3	1.393 (3)	C10—C11	1.304 (3)
C2—H2	0.9300	C10—H10	0.9300
C3—C4	1.392 (3)	C11—H11A	0.9300
C3—H3	0.9300	C11—H11B	0.9300
			
C8—N1—C1	111.23 (14)	C1—C6—C7	107.00 (15)
C8—N1—C9	123.58 (16)	O1—C7—C6	130.50 (18)
C1—N1—C9	125.10 (15)	O1—C7—C8	124.55 (17)
C2—C1—C6	120.94 (16)	C6—C7—C8	104.94 (14)
C2—C1—N1	128.18 (15)	O2—C8—N1	127.52 (18)
C6—C1—N1	110.88 (14)	O2—C8—C7	126.57 (17)
C1—C2—C3	117.64 (16)	N1—C8—C7	105.90 (15)
C1—C2—H2	121.2	N1—C9—C10	113.51 (16)
C3—C2—H2	121.2	N1—C9—H9A	108.9
C2—C3—C4	120.78 (17)	C10—C9—H9A	108.9
C2—C3—H3	119.6	N1—C9—H9B	108.9
C4—C3—H3	119.6	C10—C9—H9B	108.9
C5—C4—C3	121.93 (17)	H9A—C9—H9B	107.7
C5—C4—Br1	118.89 (13)	C11—C10—C9	126.42 (19)
C3—C4—Br1	119.16 (13)	C11—C10—H10	116.8
C6—C5—C4	116.90 (16)	C9—C10—H10	116.8
C6—C5—H5	121.5	C10—C11—H11A	120.0
C4—C5—H5	121.5	C10—C11—H11B	120.0
C5—C6—C1	121.79 (15)	H11A—C11—H11B	120.0
C5—C6—C7	131.16 (15)		
			
C8—N1—C1—C2	179.10 (18)	N1—C1—C6—C7	1.88 (19)
C9—N1—C1—C2	2.4 (3)	C5—C6—C7—O1	−0.6 (3)
C8—N1—C1—C6	−0.8 (2)	C1—C6—C7—O1	176.84 (19)
C9—N1—C1—C6	−177.57 (16)	C5—C6—C7—C8	−179.54 (17)
C6—C1—C2—C3	0.6 (3)	C1—C6—C7—C8	−2.06 (17)
N1—C1—C2—C3	−179.32 (17)	C1—N1—C8—O2	178.62 (17)
C1—C2—C3—C4	0.1 (3)	C9—N1—C8—O2	−4.6 (3)
C2—C3—C4—C5	−1.1 (3)	C1—N1—C8—C7	−0.51 (19)
C2—C3—C4—Br1	177.05 (14)	C9—N1—C8—C7	176.28 (15)
C3—C4—C5—C6	1.4 (3)	O1—C7—C8—O2	3.5 (3)
Br1—C4—C5—C6	−176.78 (12)	C6—C7—C8—O2	−177.56 (17)
C4—C5—C6—C1	−0.7 (2)	O1—C7—C8—N1	−177.40 (17)
C4—C5—C6—C7	176.46 (17)	C6—C7—C8—N1	1.59 (18)
C2—C1—C6—C5	−0.3 (3)	C8—N1—C9—C10	−90.0 (2)
N1—C1—C6—C5	179.64 (15)	C1—N1—C9—C10	86.3 (2)
C2—C1—C6—C7	−178.08 (16)	N1—C9—C10—C11	3.6 (3)

### Hydrogen-bond geometry (Å, °)

**Table:**

*D*—H···*A*	*D*—H	H···*A*	*D*···*A*	*D*—H···*A*
C2—H2···O1^i^	0.93	2.41	3.273 (2)	154
C11—H11A···O2^ii^	0.93	2.46	3.358 (3)	163

Symmetry codes: (i) *x*, *y*−1, *z*; (ii) *x*, −*y*+3/2, *z*+1/2.
